# A Fast Method to Calculate the Spatial Impulse Response for 1-D Linear Ultrasonic Phased Array Transducers

**DOI:** 10.3390/s16111873

**Published:** 2016-11-08

**Authors:** Cheng Zou, Zhenguo Sun, Dong Cai, Salman Muhammad, Wenzeng Zhang, Qiang Chen

**Affiliations:** 1 Department of Mechanical Engineering, Tsinghua University, Beijing 100084, China; zoucme@126.com (C.Z.); caid14@mails.tsinghua.edu.cn (D.C.); msb709@yahoo.com (S.M.); wenzeng@tsinghua.edu.cn (W.Z.); chenq@tsinghua.edu.cn (Q.C.); 2 Yangtze Delta Region Institute of Tsinghua University, Jiaxing 314006, China

**Keywords:** ultrasonic phased array, rectangular aperture, spatial impulse response

## Abstract

A method is developed to accurately determine the spatial impulse response at the specifically discretized observation points in the radiated field of 1-D linear ultrasonic phased array transducers with great efficiency. In contrast, the previously adopted solutions only optimize the calculation procedure for a single rectangular transducer and required approximation considerations or nonlinear calculation. In this research, an algorithm that follows an alternative approach to expedite the calculation of the spatial impulse response of a rectangular linear array is presented. The key assumption for this algorithm is that the transducer apertures are identical and linearly distributed on an infinite rigid plane baffled with the same pitch. Two points in the observation field, which have the same position relative to two transducer apertures, share the same spatial impulse response that contributed from corresponding transducer, respectively. The observation field is discretized specifically to meet the relationship of equality. The analytical expressions of the proposed algorithm, based on the specific selection of the observation points, are derived to remove redundant calculations. In order to measure the proposed methodology, the simulation results obtained from the proposed method and the classical summation method are compared. The outcomes demonstrate that the proposed strategy can speed up the calculation procedure since it accelerates the speed-up ratio which relies upon the number of discrete points and the number of the array transducers. This development will be valuable in the development of advanced and faster linear ultrasonic phased array systems.

## 1. Introduction

Ultrasonic phased array transducers are attracting attention across the board with respect to applications in both non-destructive evaluation (NDE) and clinical diagnostics. Therefore, acquiring more adequate information and enhancing inspection efficiency is significant. Ultrasound field simulation is an essential tool for probe design and optimization. The accuracy and data processing speeds are the major challenges involved in the probe design process. Various semi-analytical methods have been developed to address this problem, such as Field II [1,2], FOCUS [3,4], DREAM [5,6], Ultrasim [7], k-wave [8], and the angular spectrum technique [9,10,11,12]. Additionally, the spatial impulse response (SIR) is an excellent method to predict the transient ultrasound field radiated by an ultrasonic phased array probe in an isotropic, homogeneous, and non-dissipative medium [13]. The SIR method was first proposed by Oberhettinger [14], and then further developed by Tupholme et al. [15] and Stepanishen et al. [16,17]. Based on Huygen’s principle [17], the SIR method models the pressure field as the time-domain convolution between the first derivative of the normal vibration velocity for the transducer surface and a spatial impulse response, which is defined as a function between the observation points and the geometry of the transducer surface. Piwakowski et al. [6] proposed a method to calculate the impulse response for an arbitrarily-shaped source with arbitrary aperture velocity and delay distributions by dividing the source surface into small elements and discretizing the time variable into slices. However, the accuracy and computation time depend both on the size of the divided elements and the length of the temporal slices. For higher accuracy, it requires smaller element sizes and shorter temporal fragment lengths, which then cause a heavier computational cost. The calculation method of the spatial impulse response of a planar transducer can be simplified to avoid the labored superposition computation [17]. The SIR function is expressed as a constant multiplied with the central angle of the arc intersected between the transducer surface and a sphere with radius (*c*·*t*), which is centered at the field point *r*. Herein, *c* is the wave propagation speed in the medium and *t* is the time variable. Over the past decades, planar transducers with different geometrical shapes have been studied, such as circles [16], rectangles [13,18,19], triangles [20], and polygons [21]. Harris et al. [22] presented an excellent review on the theories and mathematical methods of transient field calculation for a baffled planar piston.

Since planar rectangular transducers are commonly employed as the elementary transmission and reception units of ultrasonic phased arrays, the calculation method of SIR of the rectangular aperture has been a popular topic. However, due to the complexity of the rectangular geometry, which involves four vertices and four edges, its calculation method for a rectangular transducer is more complicated than that for a circular planar transducer. Lockwood and Willette [23] proposed a high-speed solution for computing the SIR in the near field of a baffled planar rectangular transducer. Lee et al. [24] suggested a computationally efficient method, but it involves far field approximation. San Emeterio et al. [18] proposed a close-form description about the derivation of analytical expressions for the time-domain spatial impulse response of a rectangular piston by considering the projection position of the field point under several conditions. However, the description is complex due to the geometry discontinuity of the rectangular aperture.

Recently, many researchers have proposed the calculation of SIR for a single transducer, with a number of recently published results. Cheng et al. [19] proposed an algorithm to calculate the near and far radiation fields of a rectangular transducer by using a set of trigonometric functions which sped up the calculation by establishing a nonlinear relationship between two field points which share the same projection point on the transducer plane. The computational efficiency was improved about 20-times by incorporating their proposed nonlinear relationship in the algorithm. Alejandra et al. [13], presented a method for fast calculation of the transient near and far field of a planar rectangular piston, implementing the classic method and the approximation method using low sampling frequencies, which sped up the calculation by a factor of 3.9.

In this article, a novel and accurate computing method is proposed to further speed up the computational efficiency for the near and far fields radiated from linear ultrasonic phased array transducers. The proposed approach is based on the classical time-domain spatial impulse response approach, without any paraxial, far-field approximations, or nonlinear calculation. The derivation method for a uniformly vibrating rectangular transducer is also used and further improved with a more extensive description about the relationship between the aperture shape and the discontinuity possibilities in the temporal slope of the spatial impulse response function [18]. The aperture surface is divided into four regions, and into several sub-regions. The analytical computation is given as a set of anti-triangular functions for each sub-region.

Moreover, the state-of-the-art techniques are focused on the derivation and optimization of calculation for a single transducer. The final ultrasound field radiated from the array transducer are calculated from the summation of the field radiated from each single transducer. However, the specific characteristic of the array has never been used to speed up the calculation. Therefore, we considered the specific characteristic of the array, i.e., the shapes of the transducers in an array are assumed to be the same, meaning that the spatial relationship between the field points and different transducers are also the same, only if the relative position of the field points are identical in the local coordinate system of the transducers. With this consideration, the computational cost can be reduced if the radiated field points are elaborately designed to meet the specific characteristics of array transducers.

The main content is outlined as follows: first, the classical theory of spatial impulse response is summarized; second, the complexity of the spatial impulse response of a rectangular aperture is discussed and the relationship between the aperture shape and size, and the discontinuity possibilities, are analyzed; third, the improved computational method of the spatial impulse response for 1-D linear array transducers is discussed and, later, several computing simulations are carried out to compare the time required for the proposed method and the classical summation method.

## 2. Analytical Theory

### 2.1. Spatial Impulse Response Function

**Figure 1:**
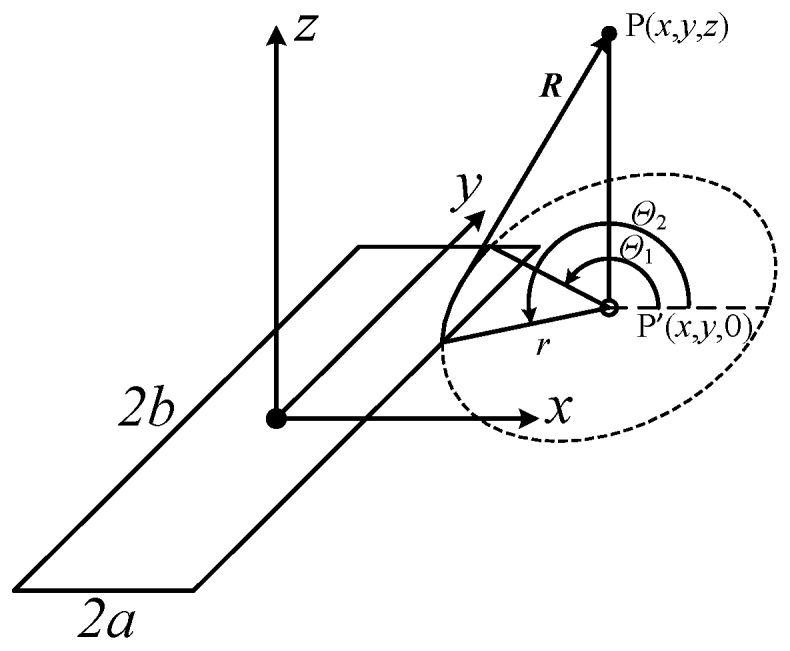
Geometry and coordinate system of the transducer and the field point.

A rectangular transducer, with length 2*b* and width 2*a* (2*b* ≥ 2*a*), is located on the plane *z* = 0, surrounded by an infinite rigid baffle, as shown in Figure 1. The radiated space is a homogeneous lossless fluid medium, with density *ρ* and speed of sound *c*. The center of the aperture is located at the origin of the coordinate system, with length along the *y* axis and width along the *x* axis. The transient pressure *p*(*x*,*y*,*z*;*t*) radiated from a planar transducer at point P(*x*,*y*,*z*), and the time instant *t*, can be written as the convolution of the first-order derivative of the normal velocity of the aperture surface, *u*(*t*) with a spatial impulse response function *h*(*x*,*y*,*z*;*t*):

(1) $$p(x,y,z;t)=\rho \frac{\partial u(t)}{\partial t}*h(x,y,z;t),$$

where the asterisk (*) denotes the convolution operation, and the spatial impulse response function *h*(*x*,*y*,*z*;*t*) is derived from the Huygens’ principle [17] as:

(2) $$h(x,y,z;t)=\int_{S}\frac{\delta (t-R/c)}{2\pi R}ds,$$

where *S* denotes the active radiating surface of the aperture, *δ*(*t*) is the Kronecker function, and *R* is the distance between the infinitesimal d*s* and the field point P(*x*,*y*,*z*).

From Equation (2), the spatial impulse response at the time instant *t* only varies with the position of the field point for a specific aperture. The surface integral in Equation (2) can be expressed instead by using polar coordinates with the origin at the field point projection on the aperture plane P′(*x*,*y*,0):

(3) $$h(x,y,z;t)=\int_{\Theta_{1}}^{\Theta_{2}}\int_{r_{1}}^{r_{2}}\frac{\delta (t-R/c)}{2\pi R}rdrd\Theta ,$$

where Θ_1_ and Θ_2_ are the minimum and maximum angle components, and *r*_2_ and *r*_1_ are the minimum and maximum radius components.

**Figure 2:**
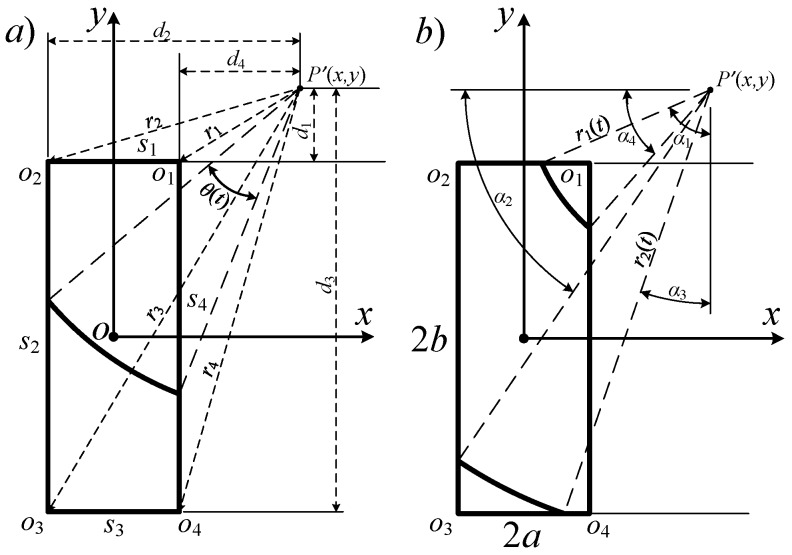
Illustration of the relevant symbols: (**a**) distance *d_i_* and *r_i_*; and (**b**) angles *α_i_* (*i* = 1–4).

Deriving from the relationship *R*^2^ = *r*^2^ + *z*^2^, the equation 2*R*d*R* = 2*r*d*r* can be obtained, and then substituting the equation with *τ* = *R*/*c* into Equation (3), the spatial impulse response function can be simplified as the integral of angle at time instant *t* multiplying with a constant term:

(4) $$\begin{array}{ll} h(x,y,z;t) & =\int_{t_{1}}^{t_{2}}\int_{\Theta_{1}(\tau )}^{\Theta_{2}(\tau )}\frac{\delta (\tau -t)}{2\pi }cd\Theta d\tau \\ & =\frac{c}{2\pi }\int_{\Theta_{1}(t)}^{\Theta_{2}(t)}d\Theta \end{array},$$

where *t*_min_ and *t*_max_ determine the time range of integration, and Θ_1_(*t*) and Θ_2_(*t*) are determined by the intersection of the transducer and the projection spherical wave centered at the field projection as shown in Figure 2 with radius *R* = *ct* at the time instant *t*. The intersection between the spherical wave and the plane *z* = 0 is a projection circle centered at P′(*x*,*y*,0) with radius $r\left(t\right)=\sqrt{{\left(ct\right)}^{2}-z^{2}}$, when *t* > *z*/*c*. The integral Equation (4) can be expressed as superimposition of the central angles of the arcs intersected between the projection circle and the transducer aperture. If more than one arc exists, the spatial impulse response can be expressed as the summation of the central angles of all the intersected arcs inside the transducer aperture:

(5) $$\begin{array}{ll} h(x,y,z;t) & =\sum_{k}\frac{c}{2\pi }\left[\Theta_{k2}(x,y,z;t)-\Theta_{k1}(x,y,z;t)\right]\\ & =\sum_{k}\frac{c}{2\pi }\Theta_{k}(x,y,z;t)\\ & =\frac{c}{2\pi }\Theta_{\Sigma }(x,y,z;t) \end{array},$$

where *k* is the index of the intersected arcs, Θ*_k_*_1_(*x*,*y*,*z*;*t*) and Θ*_k_*_2_(*x*,*y*,*z*;*t*) denote the start and the end angle of intersected arc seperately, Θ*_k_*(*x*,*y*,*z*;*t*) denotes the central angle of the *k*-th arc, and Θ_Σ_(*x*,*y*,*z*;*t*) denotes the summation of all of the central angles of the intersected arcs.

### 2.2. Spatial Impulse Response Function of a Rectangular Piston

Based on the previous derivation, the spatial impulse response function is expressed as a summation of the central angles of the intersected arcs. The calculation of the central angles become the key point for computing the spatial impulse response. Consider the Cartesian coordinates in the transducer aperture plane, as shown in Figure 2. Due to the geometrical symmetry of rectangular aperture, the projection points can be transformed to the first quadrant *x* ≥ 0, *y* ≥ 0:

(6) $$x^{\prime }=|x|,\;y^{\prime }=|y|.$$

The points in the first quadrant will be considered. The four vertices are named as *o_i_* (*i* = 1–4). The four edges are named as *s_i_* (*i* = 1–4). The distance between the projection point P′ and the vertices and the edges of the rectangle are defined as *r_i_* (*i* = 1–4) and *d_i_* (*i* = 1–4), separately:

(7) $$\begin{array}{cc} r_{1}=\sqrt{{\left(x^{\prime }-a\right)}^{2}+{\left(y^{\prime }-b\right)}^{2}}, & r_{2}=\sqrt{{\left(x^{\prime }+a\right)}^{2}+{\left(y^{\prime }-b\right)}^{2}},\\ r_{3}=\sqrt{{\left(x^{\prime }+a\right)}^{2}+{\left(y^{\prime }+b\right)}^{2}}, & r_{4}=\sqrt{{\left(x^{\prime }-a\right)}^{2}+{\left(y^{\prime }+b\right)}^{2}}, \end{array}$$

(8) $$\begin{array}{cc} d_{1}=|y^{\prime }-b|, & d_{2}=|x^{\prime }+a|,\\ d_{3}=|y^{\prime }+b|, & d_{4}=|x^{\prime }-a|. \end{array}$$

Define *td_i_* and *tr_i_* as the time constants when the projection circle expands encountering edges and the vertices:

(9) $$\begin{array}{cc} td_{i}=\sqrt{d_{i}^{2}+z^{2}}/c, & tr_{i}=\sqrt{r_{i}^{2}+z^{2}}/c \end{array}.$$

Define *t*_0_ = *z*/*c* as the spherical wave encounter with the aperture plane *z* = 0.

To reduce the calculation expressions, the angles *α_i_* (*i* = 1–4) are defined as critical angles of the intersected arc, with radius *r*(*t*) intersected with the *i*-th edge:

(10) $$\alpha_{i}=\mathrm{acos}\left[d_{i}/r(t)\right],\;\mathrm{for}\;i=1,2,3,4,$$

where *r*(*t*) denotes the radius that varies with *t* for the projection point at (*x*,*y*,*z*). The spatial coordinate symbols are omitted here. It should be noted that Equations (7) and (8) hold when *t* ≥ *t*_0_, and Equation (10) holds when the arc intersects with corresponding edge, *d_i_* ≤ *r*(*t*). The complementary angle of *α_i_* is defined as:

(11) $${\bar{\alpha }}_{i}=\frac{\pi }{2}-\alpha_{i}\;=\mathrm{asin}[d_{i}/r(t)]\;,\;\mathrm{for}\;i=1,2,3,4.$$

Due to the complexity of the rectangular aperture geometry, expression changes may occur when the intersected arcs step over the vertices or the edges of the rectangle. The expression changes then cause discontinuities in the temporal slope of Θ_Σ_(*x*,*y*,*z*;*t*) and finally affect the temporal slope of *p*(*x*,*y*,*z*;*t*). San Emeterio and Ullate [18] presented a detailed solution about this problem, and the methods proposed by them will be implemented as the classical method in the following sections. The calculation procedure varies with the position of the projection points.

**Figure 3:**
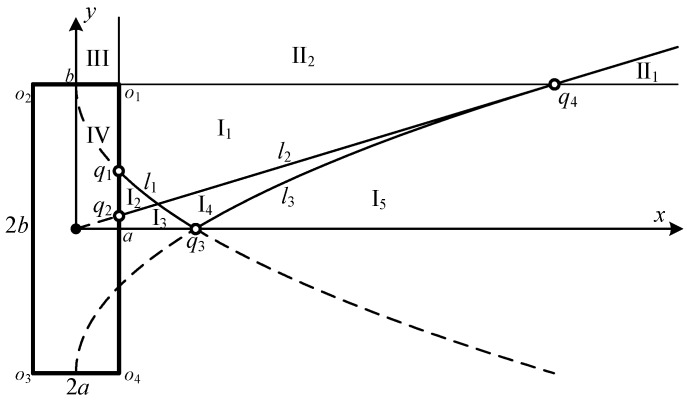
General geometry of the divided regions and sub-regions of the aperture plane.

As shown in Figure 3, the first quadrant of the aperture plane is divided into four regions by the lines *x* = *a* and *y* = *b*: I (*x* ≥ *a*, *y* ≤ *b*), II (*x* ≥ *a*, *y* ≥ *b*), III (*x* ≤ *a*, *y* ≥ *b*) and IV (*x* ≤ *a*, *y* ≤ *b*).

The curves *l*_1_, *l*_2_, and *l*_3_ denote *r*_1_ = *d*_2_, *r*_2_ = *r*_4_, and *r*_4_ = *d*_3_ separately, which are expressed as:

(12) $$\begin{array}{ccc} l_{1}: & y=b-\sqrt{4ax}, & \mathrm{when}\;a\leq x\leq b^{2}/4a,\\ l_{2}: & y=ax/b, & \mathrm{when}\;a\leq x\leq b^{2}/a,\\ l_{3}: & y=\sqrt{4ax}-b, & \mathrm{when}\;\;b^{2}/4a\leq x\leq b^{2}/a. \end{array}$$

The dashed lines are illustrated as the extension of Equation (12). The curves *l*_1_ and *x* = *a* intersect at the point *q*_1_(*a*,*b*−2*a*). The curves *l*_2_ and *x* = *a* intersect at the point *q*_2_(*a*,*a*^2^/*b*). The curves *l*_1_, *l*_3_, and *y* = 0 intersect at the point *q*_3_(*b*^2^/4*a*,0). The curves *l*_2_ and *y* = *b* intersect at the point *q*_4_(*b*^2^/*a*,*b*).

The four regions are then divided into several sub-regions by the curves. The projection points located in the same sub-region share the same calculation procedure for the central angle summation Θ_Σ_(*x*,*y*,*z*;*t*).

As presented in Figure 3, the number of sub-regions in region I relies on the position of the three intersection points *q*_1_, *q*_2_, and *q*_3_, which has direct relation to the calculation procedure of the central angle of intersected arcs. It can be derived that the position of the three intersection points are related to the ratio of the width and height of the rectangle. The relationships between the number of sub-regions in region I and the ratio of the width and height are presented in Table 1.

The order of time instant precedence and the central angle summation Θ_Σ_(*t*) for the projection points located in the sub-regions of region I are presented in Table 2. Typically, 1-D array transducers employ rectangular transducers with $a<\left(\sqrt{2}-1\right)b$, which means there are five sub-regions in the region I.

Region II is always divided into two sub-regions by the line *l*_2_, which are denoted as II_1_ and II_2_. The central angle summation calculation expressions for the projection points located in the sub-regions of region II are shown in Table 3.

Region III maintains itself without sub-regions. There is only one possibility of the discontinuity order of the time instant for the projection points located in region III. The computation procedure for projection points in region III is:

(13) $$\Theta (t)=\{\begin{array}{cc} 2\alpha_{1}, & t\in (td_{1},tr_{1})\\ {\bar{\alpha }}_{4}+\alpha_{1}, & t\in [tr_{1},tr_{2})\\ {\bar{\alpha }}_{4}+\alpha_{2}, & t\in [tr_{2},td_{3})\\ {\bar{\alpha }}_{4}+{\bar{\alpha }}_{2}-2\alpha_{3}, & t\in [td_{3},tr_{4})\\ {\bar{\alpha }}_{3}-\alpha_{2}, & t\in [tr_{4},tr_{3})\\ 0, & otherwise \end{array}.$$

**Figure 4:**
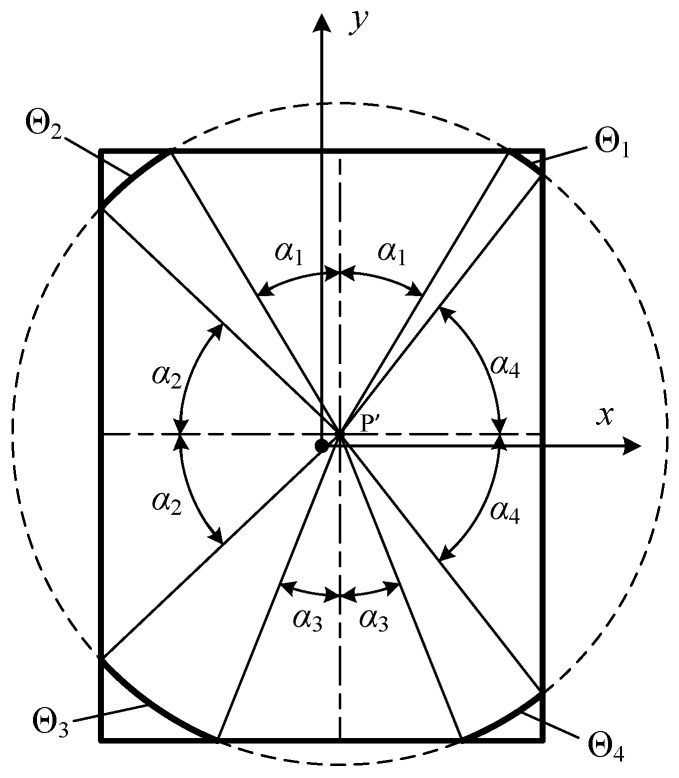
Illustration of the sub-rectangles divided from the projection point P′ and the aperture.

Since the possibilities of the discontinuity order of the time instant are complicated when the projection points are located inside the rectangle, region IV, the calculation procedure is far more complicated when compared to the procedure for points outside the rectangle [18]. The alternative solution for this condition is implemented in this paper. The projection point P′ is used to divide the rectangle aperture into four sub-rectangles, as shown in Figure 4. The shape of the rectangle, the position of the projection point P′ and the circle shown in Figure 4 are selected so as to illustrate the condition that the projection circle intersects with all of the four edges.

The final central angle summation can be expressed as the sum of central angles created by intersection of the projection circle and each sub-rectangle:

(14) $$\Theta_{\Sigma }=\sum_{k}^{4}\Theta_{k},$$

where the symbols *x*, *y*, *z*, and *t* are omitted for simplicity.

(15) $$\begin{array}{cc} \Theta_{1}=\{\begin{array}{cc} {\bar{\alpha }}_{1}-\alpha_{4}, & \mathrm{for}\;t\leq tr_{1},\\ 0, & \mathrm{for}\;t>tr_{1}, \end{array} & \Theta_{2}=\{\begin{array}{cc} {\bar{\alpha }}_{1}-\alpha_{2}, & \mathrm{for}\;t\leq tr_{2},\\ 0, & \mathrm{for}\;t>tr_{2}, \end{array}\\ \Theta_{3}=\{\begin{array}{cc} {\bar{\alpha }}_{2}-\alpha_{3}, & \mathrm{for}\;t\leq tr_{3},\\ 0, & \mathrm{for}\;t>tr_{3}, \end{array} & \Theta_{4}=\{\begin{array}{cc} {\bar{\alpha }}_{3}-\alpha_{4}, & \mathrm{for}\;t\leq tr_{4},\\ 0, & \mathrm{for}\;t>tr_{4}, \end{array} \end{array}$$

where *α_i_* is defined from Equation (10) when the arc intersects the *i*-th edge. In addition, *α_i_* equals 0 and ${\bar{\alpha }}_{i}$ equals π/2 when *r*(*t*) ≤ *d_i_*.

The expressions introduced are direct, although, they are complicated due to the complexity of the rectangular geometry. However, they can be easily implemented.

Moreover, since the intersected arcs only exist when the wave sphere intersects with the rectangular aperture, the spatial impulse response function is continuous nonzero from the first time instant, *t*_min_, the projection circle intersects the rectangle or is located within the rectangle to the last time instant, *t*_max_, the radius exceeds *r*_3_. The range of for each region and sub-region is shown in Table 4. The values beyond this range are zeros and summation procedure of these zero values can be avoided.

### 2.3. Spatial Impulse Response for Linear Array Transducers

Previously, the classical methodology for computing the transient pressure at field point (*x*,*y*,*z*) radiated from a linear array transducer with *N* planar rectangular transducers was a simple summation of the pressure from each single element [25]:

(16) $$p(x,y,z;t)=\rho \sum_{i=0}^{N-1}a_{i}h_{i}(x,y,z;t-T_{i})*\frac{\partial u_{i}(t)}{\partial t},$$

where *i* is the element index number, from 0 to *N* − 1, *h_i_*(*x*,*y*,*z*;*t*) is the spatial impulse response at field point (*x*,*y*,*z*) radiated from the *i*-th element, *u_i_*(*t*) is the aperture surface vibration velocity of the *i*-th element, and *a_i_* is the weighting level of the *i*-th element. *T_i_* is the delayed time of the *i*-th element.

Generally, the aperture surface vibration velocities are the same, so Equation (16) can be expressed as:

(17) $$p(x,y,z;t)=\rho \frac{\partial u(t)}{\partial t}*h_{\Sigma }(x,y,z;t),$$

where *h*_Σ_(*x*,*y*,*z*;*t*) is the total spatial impulse response at field point P:

(18) $$h_{\Sigma }(x,y,z;t)=\sum_{i=0}^{N-1}a_{i}h_{i}(x,y,z;t-T_{i}).$$

**Figure 5:**
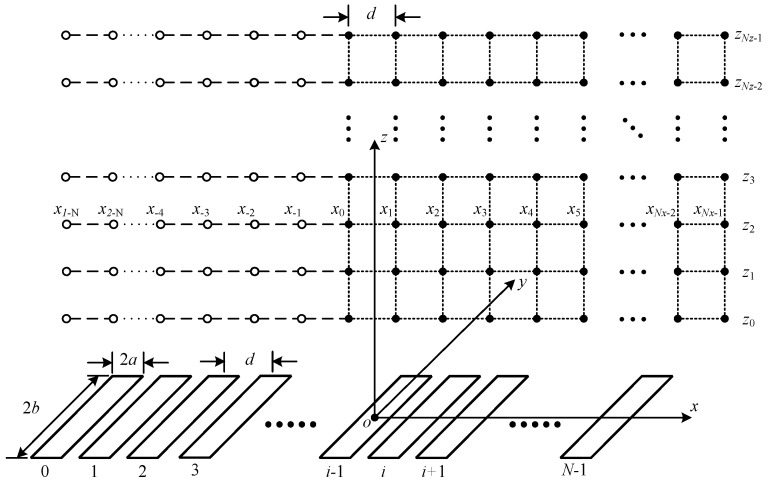
Geometry of the discretized field points and auxiliary points for linear phased array transducers.

The general geometry of linear phased array transducers and the discretized field points are shown in Figure 5.

The field points are discretized with *N_x_*, *N_y_*, and *N_z_* along with the *x*, *y*, and *z* axes. The *x* component of each point meet the relationship:

(19) $$x_{k}=x_{0}+kd,$$

where *k* is the *x* component index, and *d* is the pitch between two neighboring transducers. Although the *y* and *z* components do not have a mandatory rule, they are typically evenly distributed.

The spatial impulse response at field points (*x_k_*_+*j*_,*y*,*z*) and (*x_k_*,*y*,*z*) will meet the relationship:

(20) $$h_{i+j}(x_{k+j},y,z;t)=h_{i}(x_{k},y,z;t).$$

where *j* is an arbitrary integer.

This can be easily understood under the assumption that the geometry shape and the surface vibration velocity of the transducer elements are identical, because if two different field points have the same relative distance from two different transducers, they share the same spatial impulse response function contributed from the corresponding transducers. This means that repeated computations exist if the traditional summation method is utilized. The calculation process can be optimized for the field points which meet the distribution requirement introduced previously. The spatial impulse response *h_i_*(*x_k_*,*y*,*z*;*t*) at point (*x_k_*,*y*,*z*) contributed from transducer 1 to *N*−1 can be derived from the expression:

(21) $$h_{i}(x_{k},y,z;t)=h_{0}(x_{k-i},y,z;t),\;\mathrm{for}\;i=1,2,\cdot \cdot \cdot ,N-1,$$

which means the final result can be derived only from computing the result contributed from transducer 0. The redundant calculation procedures can be avoided.

From Equation (21), if *k* < *N* − 1, the condition that *k* − *i* < 0 may occur, which means that the right part of Equation (21) may not exist since the *x* indices of the field points are not less than zero. In order to fix this problem, *N* − 1 auxiliary points are created in the *x* direction for every *y* and *z*:

(22) $$x_{k}=x_{0}+kd,\;\mathrm{for}\;k=-1,-2,\cdot \cdot \cdot ,1-N.$$

Thus, the total spatial impulse response function at field point (*x_k_*,*y*,*z*) can be expressed as:

(23) $$\begin{array}{cc} h_{\Sigma }(x_{k},y,z;t) & =\sum_{i=0}^{N-1}a_{i}h_{i}(x_{k},y,z;t-T_{i})\\ & =\sum_{i=0}^{N-1}a_{i}h_{0}(x_{k-i},y,z;t-T_{i}) \end{array},$$

Finally, the pressure at field point (*x_k_*,*y*,*z*) can be obtained from Equation (17) by only considering the field contributed from transducer 0.

Generally, a field point gap in the *x* direction set equal to *d* lowers the obtained precision. However, if higher accuracy needs to be obtained, the proposed method can be easily modified. For more accuracy, the field point gap in the *x* direction can be set as *d*/M, where M > 1, the field points can be divided into M groups:

(24) $$x_{m,k}=x_{m,0}+kd,\;\mathrm{for}\;m=0,1,...,M-1;k=0,1,...,N_{x}-1,$$

where *x_m_*_,0_ is the start field point on direction of *x* for group *m*. Consequently, *N* − 1 auxiliary points are required to be created on direction of *x* for each group of field points:

(25) $$x_{m,k}=x_{m,0}+kd,\;\mathrm{for}\;m=0,1,...,M-1;k=-1,-2,...,1-N,$$

The spatial impulse response at field points (*x_m_*_,*k*+*j*_,*y*,*z*) and (*x_m_*_,*k*_,*y*,*z*) will meet the relationship:

(26) $$h_{i+j}(x_{m,k+j},y,z;t)=h_{i}(x_{m,k},y,z;t).$$

Correspondingly, Equation (21) can be expressed as:

(27) $$h_{i}(x_{m,k},y,z;t)=h_{0}(x_{m,k-i},y,z;t),\;\mathrm{for}\;i=1,2,\cdot \cdot \cdot ,N-1.$$

The spatial impulse response function at field point (*x_m,i_*, *y*, *z*) can be expressed as:

(28) $$\begin{array}{cc} h_{\Sigma }(x_{m,k},y,z;t) & =\sum_{i=0}^{N-1}a_{i}h_{i}(x_{m,k},y,z;t-T_{i})\\ & =\sum_{i=0}^{N-1}a_{i}h_{0}(x_{m,k-i},y,z;t-T_{i}) \end{array},$$

**Figure 6:**
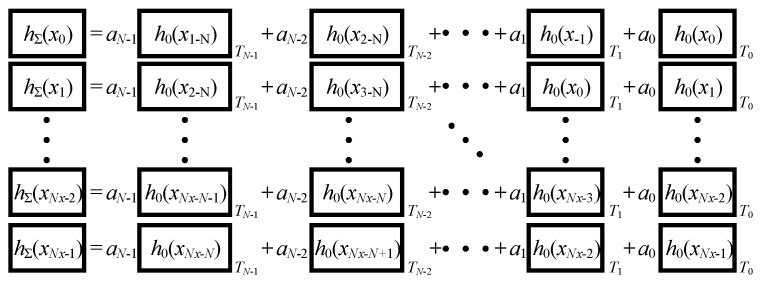
Illustration of the computation procedure for Equation (23) (symbols *y*, *z*, and *t* are omitted).

The computation procedure of Equation (28) is illustrated in Figure 6 for *m* = 0,…, M − 1 loops.

The block shown in Figure 6 denotes one step of the calculation of spatial impulse response function for a field point at the time constant *t* contributed from the one single transducer. Since the functions are related to the *x* variable, the symbols *y*, *z*, and *t* are omitted and the symbol *x_m_*_,*k*_ is simplified as *x_k_* for simplicity of illustration. *T_i_* denotes the time shift index for summation. It can be seen from Figure 6 that the calculation of the spatial impulse response involves the same transducer, which is called the basic transducer. Moreover, the basic transducer can be any one from the array transducers because the relationship shown in Equation (20) implies that the identical characteristic holds only if the field points are distributed as required.

### 2.4. Optimization of the Computation Procedure

**Figure 7:**
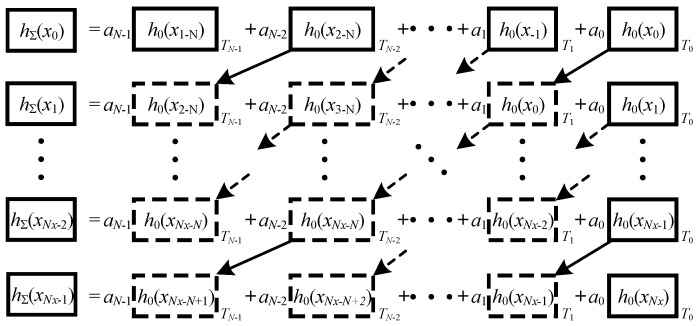
Illustration of the computation procedure for Equation (23) (symbols *y*, *z* and *t* are omitted).

Furthermore, from Figure 6, the computation procedure contains noteworthy redundancy. For example, the calculation of *h*_0_(*x*_2−N_), …, *h*_0_(*x*_−1_) and *h*_0_(*x*_0_) are used to calculate *h*_Σ_(*x*_1_) again. Only *h*_0_(*x*_1_) needs to be calculated if the results obtained during the last step can be stored in a lookup memory. Consequently, the computational cost can be reduced by reusing the results which have been calculated for the last field point. The procedure of the optimized method is illustrated in Figure 7.

The classical method for calculating the spatial impulse response for linear phased array transducers implements a simple summation of the radiated field from each transducer and suffers *N_x_* × *N_y_* × *N_z_* × *N* times the number of calculations of the spatial impulse response functions and *N_x_* × *N_y_* × *N_z_* × (*N* − 1) times the number of calculations of summations. The proposed method reduces the calculation to only (*N_x_* + *N* − 1) × *N_y_* × *N_z_* times the number of calculations of the spatial impulse response functions and *N_x_* × *N_y_* × *N_z_* × (*N* − 1) times the number of calculations of summations. The estimated speed-up ratio of computation times between the classical method and the proposed method can be expressed as:

(29) $$\begin{array}{ll} ratio & =\frac{Nx\cdot Ny\cdot Nz\cdot (N-1)}{(Nx+N-1)\cdot Ny\cdot Nz}\\ & =\frac{Nx\cdot (N-1)}{\left(Nx+N-1\right)}\\ & =\frac{1}{\frac{1}{Nx}+\frac{1}{N-1}} \end{array},$$

From Equation (29), the speed-up ratio is dependent on two factors: the number of discretized field points in the direction of the *x* axis and the transducer number. The speed-up ratio also increases along with both of the two factors.

Since the anti-trigonometric calculation process costs much more time than the summation process, this can greatly reduce the impulse response calculation process without any nonlinear approximation. Examples will be given to demonstrate how much the efficiency the proposed method can be improved. It is expected that the efficiency can be improved even more when the transducer number increases.

## 3. Simulations and Discussion

### 3.1. Comparison between the Proposed Method and the Classical Approach

In order to validate the speed-up efficiency of this new method, a series of simulations are carried out. One-dimensional linear arrays with 16, 32, 64, 128, and 256 elements are used for comparison. The arrays share the same pitch of 0.15 mm. The dimension of each rectangular transducer is the same, 0.14 mm in width and 14 mm in length. The arrays are located on the 2-D plane defined by (*x*, 0, *z*), centered at (0, 0) [*mm*] and linearly distributed along the *x* axis.

The field points are located on the 2-D plane defined by (*x*, 0, *z*). Four rectangular fields with different widths in the *x* direction and the same length, 60 mm, in the *z* direction. The points are discretized with a gap, *dx* = *dz* = 0.15 mm, in both the *x* and *z* directions. All of the fields start from *z*_0_ = 0 mm. The discretized numbers, *N_x_*, on direction of *x* are, 65, 129, 257, and 513, which form the widths of 9.6 mm, 19.2 mm, 38.55 mm, and 76.8 mm of the rectangular fields. The center of the width is at *x* = 0 mm.

The time sampling frequency is 100 MHz. The time range for calculation is from 0 to 80 μs, which involves 8001 time instants. The simulations were carried out on a Windows^©^ 7 64-bit PC with i7 4-cores 3.4 GHz CPU and 32 GB DDR3 1600 MHz memory. The proposed method and the classical method were both programed and compiled using the C++ language. The programs were established without using multithreading optimization. Both programs implemented the previously mentioned optimization algorithm to avoid redundant addition procedure of zero values.

**Figure 8:**
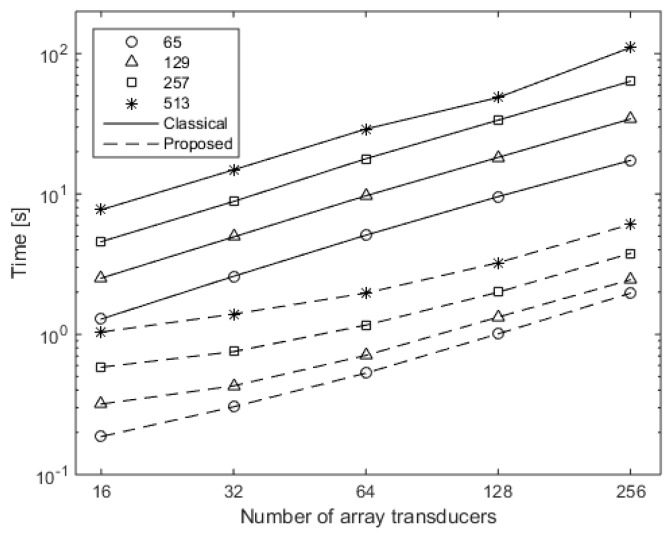
Time cost for the calculation of spatial impulse responses for different transducer numbers with different field widths, using the classical method (solid lines) and the proposed method (dashed lines).

The time cost for calculating the spatial impulse response at the 2-D plane field points radiated from the different transducers, with 16, 32, 64, 128, and 256 elements, are presented in Figure 8. The solid lines and dashed lines, with different marks, represent the results carried out using the classical method and the proposed method, respectively, for different widths of field points. Both of the axes are logarithmic coordinates for clear illustration. It can be perceived from Figure 8 that the time cost increases along with the number of the array transducers, and also along with the number of the discretized points in the direction of *x*. Additionally, compared with the results carried out using the classical method, the computation time for the proposed method has reduced. For instance, the time reduced from about 110 s to about 6 s for 256 array transducers and 513 points in the *x* direction.

**Figure 9:**
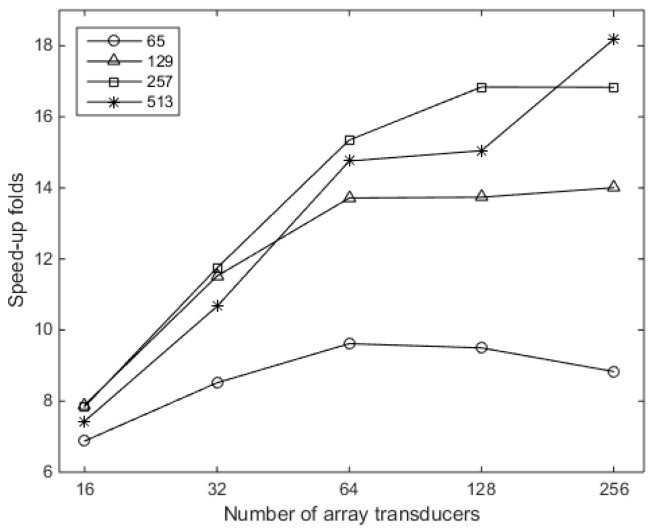
The speed-up ratios for the time cost using the proposed method in comparison with that using the classical method.

The speed-up ratio is calculated through dividing the computation time cost using the classical method by that using the proposed method for each simulation pair of the same transducer number and discretized points in the direction of *x*. The results are given in Figure 9. The speed-up ratio is both dependent on the number of the array transducers and the number of discretized point in the direction of *x*. Although, the speed-up ratio does not increase constantly along the two factors, it can be distinguished that, with more linear distributed transducers or a higher number of discretized points in the *x* direction, the speed-up ratio increases. The increment of the speed-up ratio reduces as the transducer number increases. This is due to the fact that, in the proposed method, auxiliary points are created with the same number as the transducer number in the *x* direction, which can be seen from Figure 5. This can also increase the cost calculation time if the number of auxiliary points are comparable to, or greater than, the number of the discretized points in the *x* direction. Although the speed-up efficiency will rebate under this condition, the proposed method could speed up the calculation procedure with significant ratios when the transducer number and the number of the discretized points in the *x* direction increase. For instance, the speed-up ratio reaches about 18.18 when the number of the array transducers is 256 and the number of discretized points in the *x* direction is 513.

### 3.2. Comparison between the Proposed Method and a Popular Simulation Toolbox

An example, examined in MATLAB^©^ R2016a (MathWorks, Inc., Natick, MA, USA) by using mex C++, is presented to be compared to the popular ultrasound simulation toolbox, FOCUS [3,4], which is based on fast near-field. A set of flat linear arrays with 32, 64, and 128 rectangular elements distributed along the *x*-axis is implemented. The width of the transducer element is 1 mm and the height of the transducer element is 5 mm. The spacing gap along the *x*-axis is 0.4 mm. Thus, the spacing between each neighboring transducer element is 1.4 mm. The ultrasound waves are propagated in a lossless water medium with a speed of sound of 1500 m/s. The center frequency is 1 MHz, and the sample frequency is 10 MHz. The width of the radiated field along the *x*-axis is twice that of the array width. The radiated field is discretized as a regular grid with the same spacing between the array element, *dx* = 1.4 mm. The focal point is set at (0, 0, 6λ). The radiated field is calculated by using the proposed method, based on classical spatial impulse response for each flat rectangular array element, and compared to the results calculated by using FOCUS.

**Figure 10:**
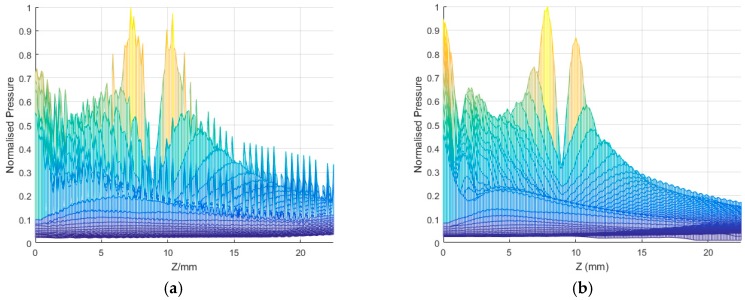
The field radiation along the *z*-axis at x = 0 and y = 0, with 64 array elements. (**a**) Calculated by using the proposed method; and (**b**) calculated by using FOCUS.

Figure 10 shows an example in which the results calculated by using the proposed method is compared with a popular simulation toolbox, FOCUS, which is based on far near field method. The simulation condition is described previously. The accuracy of the spatial impulse response depends on the sampling time. In this example, only 10 times that of the center frequency is used for sampling. It can be seen that FOCUS has a good result in accuracy when using a lower sampling frequency, which is the main contribution of FOCUS. In order to obtain a more accurate result, the classical method of spatial impulse response requires a higher sampling frequency. However, this does not affect the acceleration ability since the proposed method is frequency independent as long as the method used to calculate each single transducer only depends on the relative spatial position between the field points and the center of array transducer.

The time cost to calculate the result for different array element numbers, respectively using the proposed method and FOCUS, is presented in Table 5. Since the time cost depends both on the method, intrinsically, and also on the programming methodology, the comparison is just qualitative so as to show that the proposed method can effectively reduce the calculation time. It can be seen that the quantity of repeating calculations seriously increases if the hidden redundant calculation steps are not taken into account, especially when array aperture expands. However, the time cost by using the proposed method increases marginally.

Since the proposed speed-up method is based on solving the repeating calculation expressions which only depends on the relative position between the space field point and the array element, any method conforming to this principle can be used as the method to calculate the wave field radiated from each transducer.

**Figure 11:**
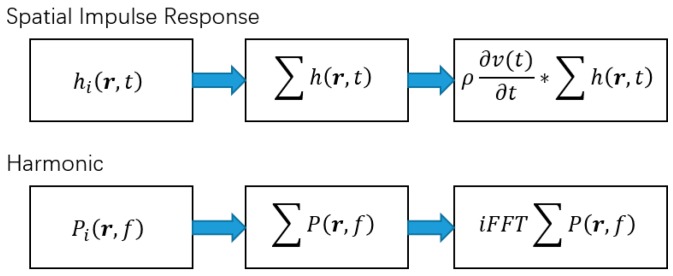
The main calculation steps for the transient radiated field.

The repeating of array transducers becomes crucial when the array aperture expands. The previous calculation methods can be divided into two approaches, as illustrated in Figure 11. First, like the spatial impulse response, the impulse response, which is dependent on the relative spatial position, was calculated and then the transient radiated field is calculated from the convolution between the superimposed impulse response and the first derivation of the impulse signal as expressed in Equation (17). Second, like FOCUS, which calculates the harmonic wave field for a series of frequencies, and then calculates the transient field by using the inverse Fourier transform at each field point. Both methods involving the spatial impulse response or harmonic waves are dependent on the spatial relative position between the field points and each array transducer. As described previously, the repeating calculation is hidden if the field grid is discretized casually, which can be solved by using a specific regular grid method as presented in this paper. The proposed method can be beneficial for any other analytical or semi-analytical calculation method in which the main steps depend on the relative spatial position between field points and the array elements, no matter whether or not it breaks the transducer elements into subsections or has any approximation for the single element.

Intrinsically, the proposed method does not change the calculation steps for each single transducer, although it improves the calculation steps, so the accuracy depends just on the calculation method of a single transducer element and the sampling frequency.

On the other hand, a parallel computing step is also hidden in the first step and the last step, which is not referred to in this paper, however this can also be a beneficial tool to improve the calculation speed by designing an effective parallel program by using multi-core computer.

Moreover, other than the advantages of the proposed method described previously, it, in itself, does have some disadvantages. First, the proposed method cannot deal with imaging problems [26], since the imaging functions are non-linear related to spatial components. Second, the proposed method cannot deal with irregular grids that do not conform to the distribution rule of the array elements, since regular grids are the key to solving the repeating calculation problem. Although it has some limitations, it is accurate enough if the grid spacing is equal or less than the element spacing, since the majority of array elements are distributed with comparative spacing to the wavelength.

## 4. Conclusions

A method is proposed to compute the spatial impulse response for 1-D linear phased array transducers. This method is based on the classical spatial impulse response calculation method for rectangular transducers, without aperture discretization and superposition, for rectangular apertures. The rectangular aperture surface is divided into several sub-regions. The relationship between the possibilities of discontinuities and the geometry of the aperture is proposed. The spatial impulse response functions are derived for each sub-region. Under the assumption that the transducer elements have the same rectangular geometry, an analytical expression is derived for the spatial impulse responses from two different field points sharing the same relative distance from two different transducers. Simulation with different numbers of array transducers for different dimensions of field points have been carried out. The time cost for the calculation of the spatial impulse response is reduced by implementing the proposed method. The speed-up ratio varies with the number of array transducers, and also the number of discretized points on direction of *x*. Since this method is based on analytical derivation without approximation or nonlinear transformation, the results are accurate. The effort to solve the repeating calculation problem regarding the spatial-dependent expressions is important because unnecessary calculations can seriously reduce the calculation speed.

Moreover, it may be beneficial if combined with other speed-up methods, for instance, the approximation method proposed in [13] or the nonlinear transformation in the *z*-axis direction proposed in [19]. This method may have potential to improve the design speed of ultrasound linear phased array systems.

## Figures and Tables

**Table 1:** Relationship between the rectangular geometry and the possibility of discontinuities.

Relationship of Width and Height	Sub-Regions
$a<\left(\sqrt{2}-1\right)b$	5
$\left(\sqrt{2}-1\right)b\leq a<b/2$	4
*b*/2 ≤ *a* < *b*	3

**Table 2:** Central angle summation of the intersected arc for projection points located in sub-regions I_1_, I_2_, I_3_, I_4_, and I_5_.

I_1_		I_2_		I_3_		I_4_		I_5_	
*t*	Θ_Σ_(*t*)	*t*	Θ_Σ_(*t*)	*t*	Θ_Σ_(*t*)	*t*	Θ_Σ_(*t*)	*t*	Θ_Σ_(*t*)
(*td*_4_,*tr*_1_)	$2\alpha_{4}$	(*td*_4_,*td*_2_)	$2\alpha_{4}$	(*td*_4_,*td*_2_)	$2\alpha_{4}$	(*td*_4_,*tr*_1_)	$2\alpha_{4}$	(*td*_4_,*tr*_1_)	$2\alpha_{4}$
[*tr*_1_,*td*_2_)	${\bar{\alpha }}_{1}+\alpha_{4}$	[*td*_2_,*tr*_1_)	$2\alpha_{4}-2\alpha_{2}$	[*td*_2_,*tr*_1_)	$2\alpha_{4}-2\alpha_{2}$	[*tr*_1_,*td*_2_)	${\bar{\alpha }}_{1}+\alpha_{4}$	[*tr*_1_,*tr*_4_)	${\bar{\alpha }}_{1}+\alpha_{4}$
[*td*_2_,*tr*_2_)	${\bar{\alpha }}_{1}+\alpha_{4}-2\alpha_{2}$	[*tr*_1_,*tr*_2_)	${\bar{\alpha }}_{1}+\alpha_{4}-2\alpha_{2}$	[*tr*_1_,*tr*_4_)	${\bar{\alpha }}_{1}+\alpha_{4}-2\alpha_{2}$	[*td*_2_,*tr*_4_)	${\bar{\alpha }}_{1}+\alpha_{4}-2\alpha_{2}$	[*tr*_4_,*td*_2_)	${\bar{\alpha }}_{1}+{\bar{\alpha }}_{3}$
[*tr*_2_,*tr*_4_)	$\alpha_{4}-\alpha_{2}$	[*tr*_2_,*tr*_4_)	$\alpha_{4}-\alpha_{2}$	[*tr*_4_,*tr*_2_)	${\bar{\alpha }}_{1}+{\bar{\alpha }}_{3}-2\alpha_{2}$	[*tr*_4_,*tr*_2_)	${\bar{\alpha }}_{1}+{\bar{\alpha }}_{3}-2\alpha_{2}$	[*td*_2_,*tr*_2_)	${\bar{\alpha }}_{1}+{\bar{\alpha }}_{3}-2\alpha_{2}$
[*tr*_4_,*tr*_3_)	${\bar{\alpha }}_{3}-\alpha_{2}$	[*tr*_4_,*tr*_3_)	${\bar{\alpha }}_{3}-\alpha_{2}$	[*tr*_2_,*tr*_3_)	${\bar{\alpha }}_{3}-\alpha_{2}$	[*tr*_2_,*tr*_3_)	${\bar{\alpha }}_{3}-\alpha_{2}$	[*tr*_2_,*tr*_3_)	${\bar{\alpha }}_{3}-\alpha_{2}$
*otherwise*	0	*otherwise*	0	*otherwise*	0	*otherwise*	0	*otherwise*	0

**Table 3:** Central angle summation of the intersected arc for projection points located in sub-regions II_1_ and II_2_.

II_1_		II_2_	
*t*	Θ(*t*)	*t*	Θ(*t*)
(*tr*_1_,*tr*_4_)	$\alpha_{4}-{\bar{\alpha }}_{1}$	(*tr*_1_,*tr*_2_)	$\alpha_{4}-{\bar{\alpha }}_{1}$
[*tr*_4_,*tr*_2_)	$\alpha_{1}-\alpha_{3}$	[*tr*_2_,*tr*_4_)	$\alpha_{4}-\alpha_{2}$
[*tr*_2_,*tr*_3_)	${\bar{\alpha }}_{3}-\alpha_{2}$	[*tr*_2_,*tr*_3_)	${\bar{\alpha }}_{3}-\alpha_{2}$
*otherwise*	0	*otherwise*	0

**Table 4:** Time instant range, [*t*_min_, *t*_max_], for each region/sub-region.

Regions (Sub-Regions)	*t*_min_	*t_max_*
I (I_1_, I_2_, I_3_, I_4_, I_5_)	*td*_4_	*tr*_3_
II (II_1_, II_2_)	*tr*_1_	
III	*td*_2_	
IV	0	

**Table 5:** Time cost by using the proposed method and FOCUS, respectively, for different array apertures (seconds).

Array Element	Proposed Method	FOCUS
16	0.0226	0.15847
32	0.0636	0.70662
64	0.1794	10.1498
128	0.6708	132.8804
256	2.3405	1276.9548

## References

[1] Jensen J.A. (1996). Field: A Program for simulating ultrasound systems. Med. Biol. Eng. Comput..

[2] Jensen J.A., Svendsen N.B. (1992). Calculation of pressure fields from arbitrarily shaped, apodized, and excited ultrasound transducers. IEEE Trans. Ultrason. Ferroelectr..

[3] McGough R.J. (2004). Rapid calculations of time-harmonic nearfield pressures produced by rectangular pistons. J. Acoust. Soc. Am..

[4] McGough R.J., Samulski T.V., Kelly J.F. (2004). An efficient grid sectoring method for calculations of the near-field pressure generated by a circular piston. J. Acoust. Soc. Am..

[5] Piwakowski B., Sbai K. (1999). A new approach to calculate the field radiated from arbitrarily structured transducer arrays. IEEE Trans. Ultrason. Ferroelectr..

[6] Piwakowski B., Delannoy B. (1989). Method for computing spatial pulse response: Time-domain approach. J. Acoust. Soc. Am..

[7] Holm S. Simulation of Acoustic Fields from Medical Ultrasound Transducers of Arbitrary Shape. Proceedings of the Nordic Symposium in Physical Acoustics.

[8] Treeby B.E., Cox B.T. (2010). k-Wave: MATLAB toolbox for the simulation and reconstruction of photoacoustic wave fields. J. Biomed. Opt..

[9] Belgroune D., de Belleval J.F., Djelouah H. (2008). A theoretical study of ultrasonic wave transmission through a fluid–solid interface. Ultrasonics.

[10] Song S., Kim C. (2002). Simulation of 3-D radiation beam patterns propagated through a planar interface from ultrasonic phased array transducers. Ultrasonics.

[11] Belgroune D., de Belleval J.F., Djelouah H. (2002). Modelling of the ultrasonic field by the angular spectrum method in presence of interface. Ultrasonics.

[12] Talbi R., Assaad J., Rouvaen J., Boudy P., Haine F. (1998). Application of the angular spectrum approach to compute the far-field pressure. Ultrasonics.

[13] Ortega A., Tong L., D’hooge J. (2014). A new analytic expression for fast calculation of the transient near and far field of a rectangular baffled piston. Ultrasonics.

[14] Oberhettinger F. (1961). On transient solutions of the baffled piston problem. J. Res. Natl. Inst. Stan..

[15] Tupholme G.E. (1969). Generation of acoustic pulses by baffled plane pistons. Mathematika.

[16] Stepanishen P.R. (1971). Transient radiation from pistons in an infinite planar baffle. J. Acoust. Soc. Am..

[17] Stepanishen P.R. (1971). The time-dependent force and radiation impedance on a piston in a rigid infinite planar baffle. J. Acoust. Soc. Am..

[18] San Emeterio J.L., Ullate L.G. (1992). Diffraction impulse response of rectangular transducers. J. Acoust. Soc. Am..

[19] Cheng J., Lu J., Lin W., Qin Y. (2011). A new algorithm for spatial impulse response of rectangular planar transducers. Ultrasonics.

[20] Jensen J.A. (1996). Ultrasound fields from triangular apertures. J. Acoust. Soc. Am..

[21] Jensen J.A. (1999). A new calculation procedure for spatial impulse responses in ultrasound. J. Acoust. Soc. Am..

[22] Harris G.R. (1981). Review of transient field theory for a baffled planar piston. J. Acoust. Soc. Am..

[23] Lockwood J.C., Willette J.G. (1973). High-speed method for computing the exact solution for the pressure variations in the nearfield of a baffled piston. J. Acoust. Soc. Am..

[24] Lee C., Benkeser P.J. (1994). A computationally efficient method for the calculation of the transient field of acoustic radiators. J. Acoust. Soc. Am..

[25] Ullate L.G., San Emeterio J. (1992). A new algorithm to calculate the transient near-field of ultrasonic phased arrays. IEEE Trans. Ultrason. Ferroelectr..

[26] Schmerr L.W. (2015). Fundamentals of Ultrasonic Phased Arrays.

